# Private costs of carbon emissions abatement by limiting beef consumption and vehicle use in the United States

**DOI:** 10.1371/journal.pone.0261372

**Published:** 2022-01-19

**Authors:** Brandon R. McFadden, Paul J. Ferraro, Kent D. Messer

**Affiliations:** 1 University of Delaware, Newark, Delaware, United States of America; 2 Johns Hopkins University, Baltimore, Maryland, United States of America; University of Naples Federico II: Universita degli Studi di Napoli Federico II, ITALY

## Abstract

A popular strategy for mitigating climate change is to persuade or incentivize individuals to limit behaviors associated with high greenhouse gas emissions. In this study, adults in the mid-Atlantic United States bid in an auction to receive compensation for eliminating beef consumption or limiting vehicle use. The auction incentivized participants to reveal their true costs of accepting these limits for periods ranging from one week to one year. Compliance with the conditions of the auction was confirmed via a random field audit of the behavioral changes. The estimated median abatement costs were greater than $600 per tCO2e for beef consumption and $1,300 per tCO2e for vehicle use, values much higher than the price of carbon offsets and most estimates of the social cost of carbon. Although these values may decline over time with experience or broader social adoption, they imply that policies that encourage innovations to reduce the costs of behavior change, such as meat alternatives or emission-free vehicles, may be a more fruitful than those that limit beef consumption or vehicle use.

## Introduction

In a new book, Bill Gates advises the public about various sources of greenhouse gases (GHGs) emissions and how changes in consumption can help achieve zero emissions and limit anthropogenic climate change [1]. Gates recommends four changes that consumers can make: try a plant-based burger, buy an electric vehicle, reduce home emissions, and opt in to a green pricing program with an electricity provider [1]. Encouraging individuals to reduce the frequency of their high-emission choices is a common theme among climate change mitigation advocates [2] because an individual’s carbon footprint is a function of lifestyle choices [3, 4].

While fewer high-emission choices by just one individual would have no measurable impact on GHG emissions, fewer high-emission choices by many could have a large impact. Yet coordination across large portions of society is unlikely, unless changes in consumption are highly visible to others and there is a high level of in-group trust that others will be reciprocators [5]. It follows that focus should be given to *both* high-emission choices and consumption behaviors that can be adopted and sustained by broad groups of society.

The McKinsey Marginal Abatement Cost Curve was developed to communicate the relative abatement potential and cost associated with possible behavioral changes so government and industry decision-makers could prioritize efficient, cost-effective strategies to reduce GHG emissions [6]. The construction of a similar framework for individual-level abatement would be insightful for consumers and society.

To prioritize choices or behaviors to target for policy intervention, *plasticity* should be considered along with abatement potential. Plasticity is a construct used by academics to measure the proportion of nonadopters willing to change a choice or behavior [7]. For example, the proportion of meat eaters who could be induced to become vegetarians. Policy interventions are likely to provide the best return-on-investment when they target choices and behaviors for which abatement potential *and* plasticity are high enough to lead to meaningful reductions in GHG emissions.

The individual behavior with the largest impact on GHG emissions is the choice to have a child [3]. However, its plasticity is low: coercing or incentivizing individuals to reduce fertility is likely to be costly, difficult to enforce, and controversial. Therefore, plans to address climate change generally target behaviors with higher plasticity, such as food and travel choices. Substantial GHG emissions are associated with food and travel choices [8–10], and these choices may be less costly, less controversial, and more straightforward to change than childbearing choices.

Recommendations to reduce emissions associated with travel have a long history. For example, President Clinton’s Climate Change Action Plan in 1993 recommended innovative strategies to reduce vehicle miles traveled [11]. More recently, dietary changes to reduce emissions and other social costs have also been recommended and received considerable attention, as groups like the EAT-Lancet Commission recommended a diet with little or no red meat [12]. Although, the diet recommended by the commission is not economically sustainable as more than 1.58 billion people could not afford to follow the EAT–Lancet diet even if they allocated all household income to their food budget [13]. Bill Gates acknowledged this In an interview with MIT Technology Review while discussing his new book, stating “So no, I don’t think the poorest 80 countries will be eating synthetic meat. I do think all rich countries should move to 100% synthetic beef.” [14].

Although studies have examined how taxes, as a policy intervention, affect food and travel choices [15–18], we know of no study that estimates the private costs of abating GHGs from [food and travel] choices. Prior studies report that food consumption overall is unresponsive (inelastic) to increases in sin taxes [19] but beef consumption did decline when taxes on the GHG content of food were applied in the EU [20]. Yet GHG taxes are likely to be disproportionally borne by lower-income households [21]. Moreover, GHG taxes have unintended consequences that can be difficult to control. For example, GHG taxes will increase the consumption of other meat like chicken and pork if consumers are more willing to substitute those products than absorb the change in beef price [20]. Also, foods that have relatively lower emissions may be unhealthy [22], as nutritious diets are not necessarily associated with relatively lower emissions [23]. If prices are a function of GHG emissions, informing consumers that higher prices are due to emissions may backfire and increase in the probability of choosing a higher-emitting option [24]. To start to understand these tradeoffs, more information on the private costs of reducing beef and fuel consumption is needed.

In this study, we estimate the private costs of reducing beef and fuel consumption across various time periods (i.e., one week, one month, three months, six months, and one year) among adults in the Mid-Atlantic United States. A non-hypothetical auction was used to incentivize participants to offer their level of compensation necessary to eliminate beef from their diets or limit vehicle miles traveled for each length of time. The cash payment for participants who ‘won’ the auction was conditional on passing an audit to assess compliance with the targeted behavior changes.

Combining the abatement potential of each behavior with elicited offers map out abatement cost curves. These curves allow us to compare the estimated abatement costs associated with eliminating beef consumption and limiting vehicle travel, as well as to compare the costs of these behavior changes with the abatement costs of other actions (e.g., offsets) and the social cost of carbon. After an initial analysis of data for all participants, we present the abatement costs and abatement potential associated with a plasticity target of 50%, which corresponds to the point at which approximately half of the sample with the lowest offers engages in a behavior change. To provide insight into the variability of abatement costs associated with different plasticity targets, we also consider plasticity targets of 25% and 75% which correspond to the offers within the 25^th^ and 75^th^ percentiles changing behavior.

## Materials and methods

This study was approved by the University of Delaware IRB. Written consent was obtained, and this study did not include minors. A pre-analysis plan of this field study was registered on Open Science Framework prior to the launching of the auction (available at: https://osf.io/x2yrj/?view_only=aa2328a1d4004f659157abeed847d734). Data were collected from April 27 to May 21, 2019.

### Auction design

Participants revealed individual costs, referred to as their “offers,” of reducing beef consumption and personal vehicle travel for five time-horizons: one week, one month, three months, six months, and one year. Offers were elicited using a reverse auction (i.e., the minimum amount of money required to change their behavior), which has previously been used by economists to elicit individual costs associated with a behavioral change [25–27].

GHG emissions from personal vehicle use is much higher than beef consumption, at least for the average consumers. Therefore, participant offers were based on eliminating beef from a diet and limiting vehicle use so that costs necessary to induce changes in these behaviors could be compared more directly. Miles traveled were capped at 200, 875, 2625, 5250, and 10,500 miles depending on the length of time. The caps were chosen so that abatement potential for eliminating beef and limiting fuel consumption were approximately equal for the average consumer and vehicle fuel efficiency in the US (these limits correspond to a reduction in miles of about 16%, on average; see Methods for details).

The amount of compensation required for participants to change behavior was elicited by two questions: (1) What is the lowest dollar amount for which you would be willing to stop eating beef for one week [one month, three months, six months, one year]? (2) What is the lowest dollar amount for which you would be willing to limit driving your personal vehicle(s) to no more than 200 [875, 2,625, 5,250, 10,500] miles for one week [one month, three months, six months, one year]?” The order in which the questions were presented was randomized across participants to reduce any order effects associated with their offers. Each participant submitted ten independent offers (two behaviors, five lengths of time).

Compensation for the participants who ‘won’ was determined using a second-price auction, which provides incentive compatibility because participants who offer a value different from their true reservation price (i.e., cost) are penalized [28]. In a second-price auction, the participant who required the least compensation to make a behavioral change ‘won’ and were compensated the amount of the second-lowest offer. To facilitate understanding of the auction rules and maintain consistency of information provided, a video was presented to all participants that explained how a ‘winner’ would be chosen and why it is in the participant’s best interest to always reveal the value equal to their lowest required compensation. The video explained simple scenarios in which a person under-reports and over-reports their true lowest compensation required to illustrate that a participant would be worse off by offering a value different from their true reservation cost.

### Auction compliance

**Fig 1:**
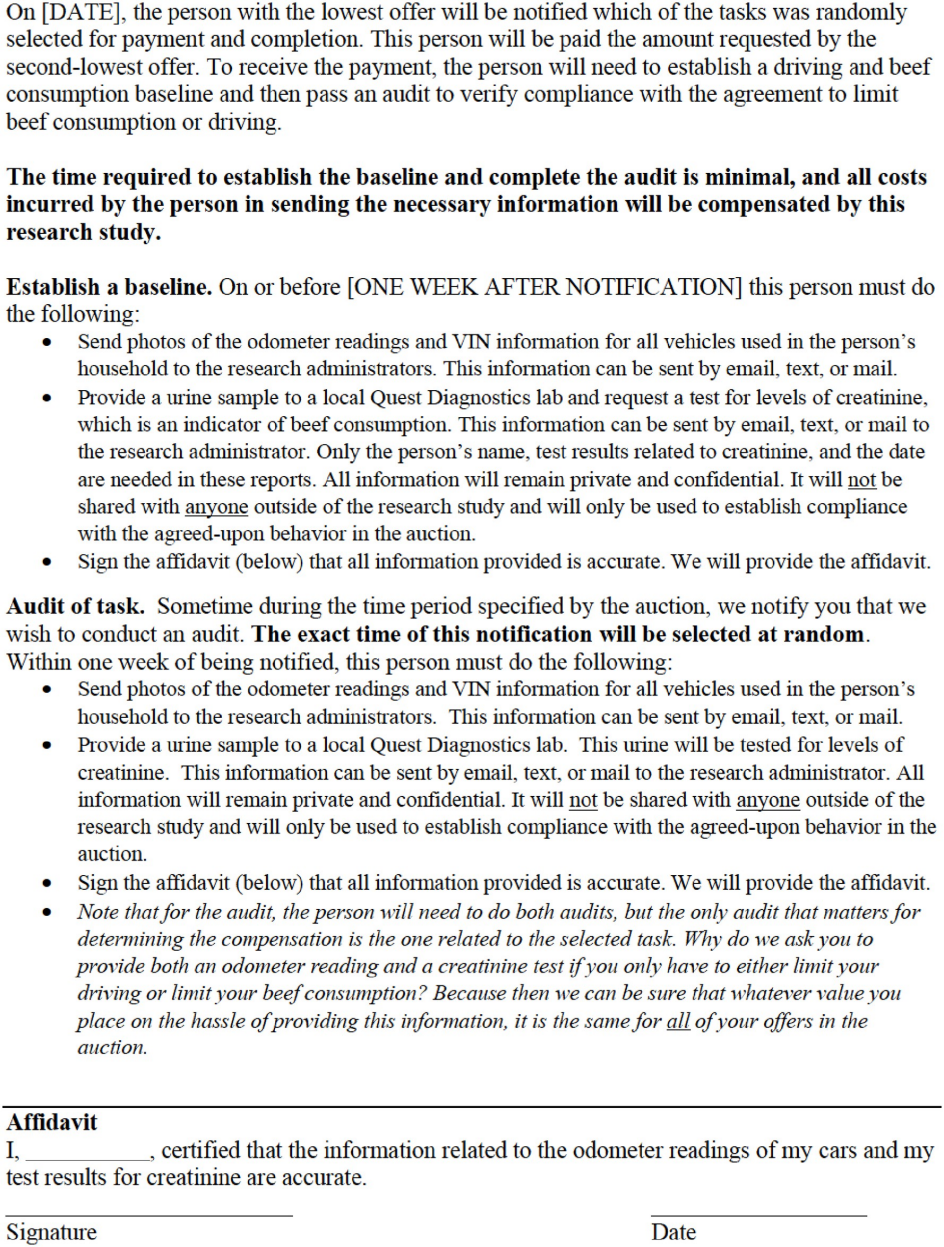
Affidavit describing the audit and signed by participants.

The consent form provided to participants prior to the auction communicated that by consenting to take part in the study they also agree to complete an audit for the behavioral changes if selected as a ‘winner,’ and participants were informed that a ‘winner’ of the auction would be determined from one of the five time-horizons randomly selected by the research team. The audit included providing two urine samples to a local diagnostics lab and provide two photos of their car and odometer. After consenting to take part in the study, participants were asked to sign an affidavit to comply with the audit. An image of the affidavit is shown in Fig 1. Participants were informed that costs incurred to complete the audit would be covered by the study. and participants were asked about the perceived cost of complying with the audit, and they reported that these costs did not substantially affect offers. The first urine sample and photos were provided by a ‘winner’ to establish a baseline, and the second sample and photo were to be provided during the randomly selected time horizon. Participants were informed that the exact time the second sample and photo were to be provided would be selected at random by the research team; they were given a week to provide the second sample and photo.

The auction compliance was enforced to minimize the hypothetical bias associated with eliciting values from individuals. Nevertheless, if participants with high reservation costs believed there was little chance of winning the auction, hypothetical bias might be a concern. However, no evidence of hypothetical bias was found in a recent meta-analysis of comparisons of real versus hypothetical willingness-to-accept elicitation procedures [29].

### Calculation for vehicle use limits

A recent life cycle assessment of US beef estimated that direct GHG emissions are 21.3 kg of carbon dioxide emissions (kgCO_2_e) per kg of carcass weight [30], which is similar to earlier estimates of 22 kg [31]. The disappearance of beef in the US, which is the fresh and processed meat sold through grocery stores and used in restaurants, was 37.06 kg per capita in 2018 (“disappearance” is a term of art in the agricultural sector, and its value provides a reliable estimate of annual human consumption) [32]. Using the estimates of direct emissions and disappearance, per capita GHG emissions from beef consumption in the US was 789 kgCO_2_e in 2018.

**Fig 2:**
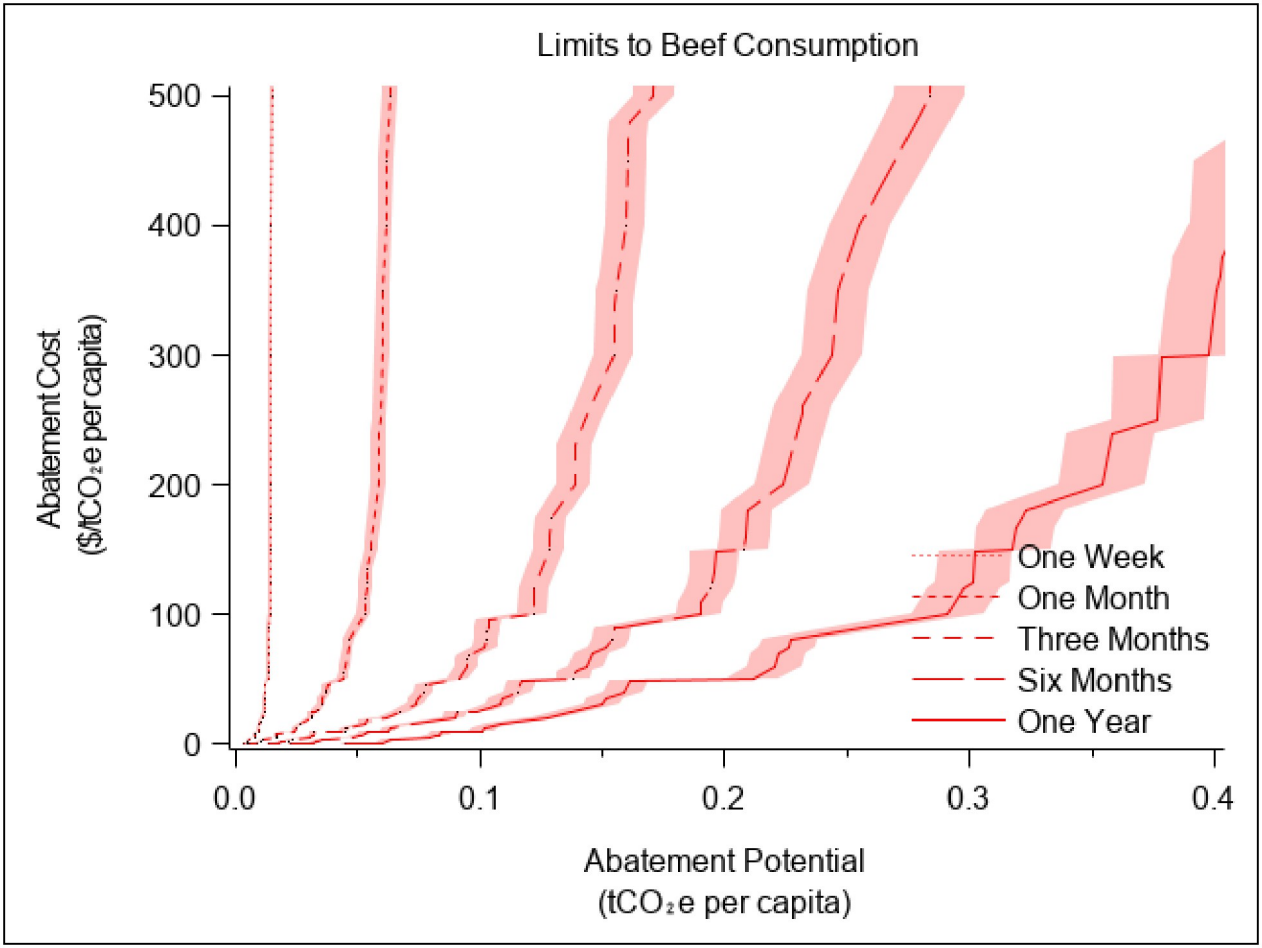
Revealed abatement cost curves to eliminate beef consumption. The lines represent the amount of money ($US) required to abate carbon emissions by eliminating beef consumption for different time periods. The figure presents cost curves for a plasticity target of approximately 50% (i.e., costs are a function of the offer values at or below the median offer). The error bands bound the potential changes in costs as the assumption about status quo beef consumption changes by plus or minus 5%.

**Fig 3:**
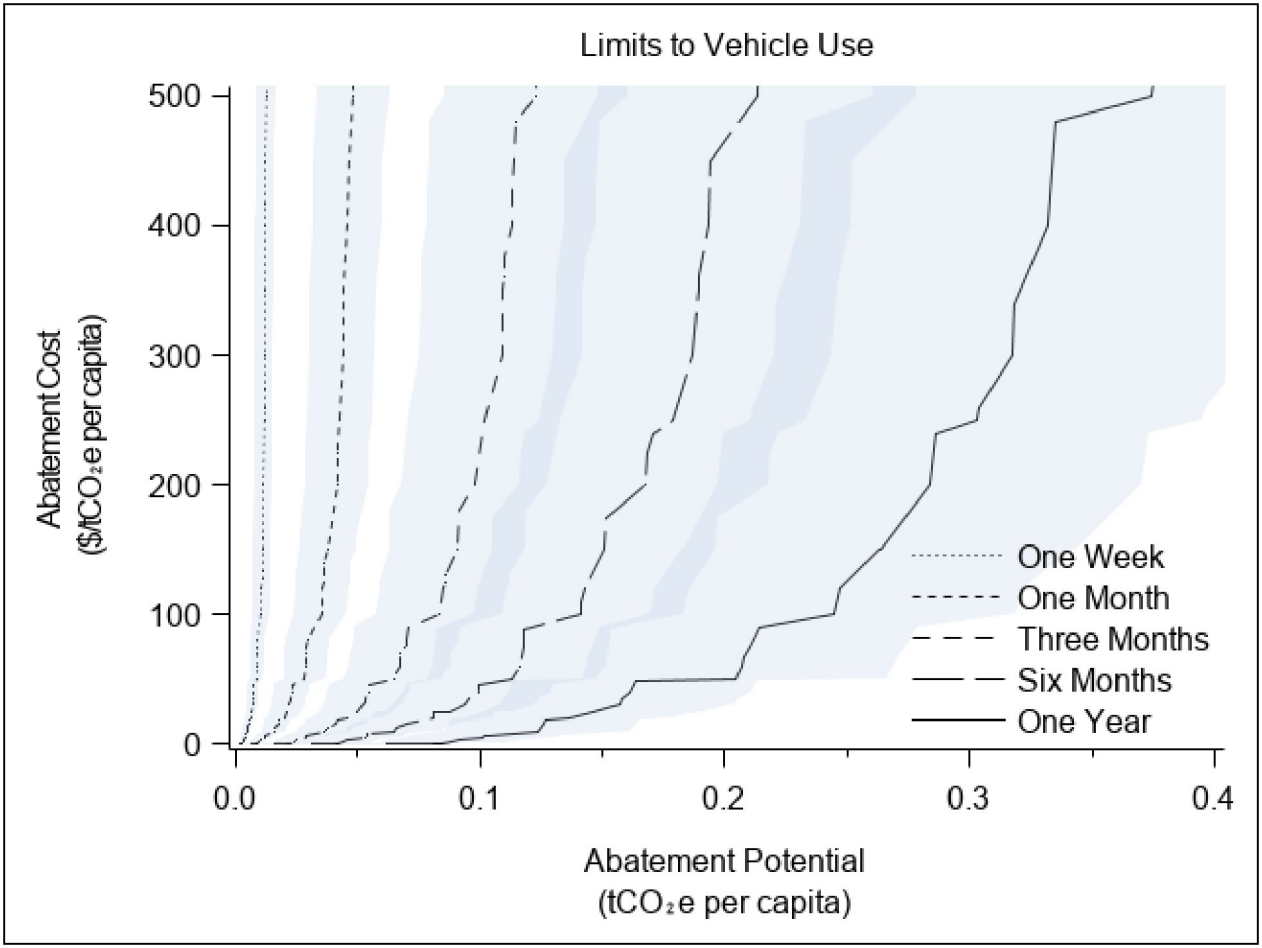
Revealed abatement cost curves to limit personal vehicle use. The lines represent the amount of money ($US) required to abate carbon emissions by limiting personal vehicle use for different time periods. The figure presents cost curves for a plasticity target of approximately 50% (i.e., costs are a function of the offer values at or below the median offer). The error bands bound the potential changes in costs as the assumption about status quo driving levels changes by plus or minus 5%.

According to the US Department of Transportation, in 2017 the average number of vehicle miles traveled per capita annually in the mid-Atlantic state where the study was conducted was 11,657 and the average annual miles driven per capita nationwide was 13,476 [33, 34]. The US Environmental Protection Agency estimates that average fuel economy for US cars is 24.7 miles per gallon [35], and the US Energy Information Administration estimates that the emissions from a gallon of gasoline is 8.89 kgCO_2_e [36]. Using a simple average of the US Department of Transportation estimates of per capita vehicle use (12,567 miles), we calculate that use of a vehicle annually generates emissions of 4,523 kgCO_2_e and that GHG emissions from beef consumption are approximately 17% of GHG emissions from vehicle use. To provide the same 789 kgCO_2_e annual reduction in emissions estimated for beef, individuals could reduce their driving by 2,193 miles per year (from an average of 12,567 to an average of 10,373). In the study, we increased the overall reduction slightly from 10,373 to 10,500 miles to make it easier for participants to envision changes in their driving. The values used to determine the mileage caps are shown in S7 Table in S1 File. In our sensitivity analyses in Figs 2 and 3 and S1-S5 Figs in S1 File, we changed the assumed consumption levels by plus and minus 5%.

### Participants and covariates

Our convenience sample included 626 participants recruited at a public event or while visiting a retail outlet at a large public university in the mid-Atlantic region of the US. Note that, in a review of behavioral studies related to climate change mitigation, none of the meat consumption studies had more than 500 participants and over 80% of the studies had fewer than 100 participants [37]. To qualify for the study, participants were required to be over the age of 21 (confirmed with ID), have consumed beef (self-reported), and own a personal vehicle (self-reported). Participants received a $10 participation fee and were also screened to prevent multiple offers by the same individual.

While most of the participants came from the mid-Atlantic states of Delaware, Maryland, New Jersey, New York, Pennsylvania, and Virginia, the sample also include some participants from Arizona, California, Colorado, Connecticut, Florida, Indiana, Kentucky, Louisiana, Massachusetts, Michigan, North Carolina, Vermont, and Washington. The median age category of participants was 25–34, the median income category was US$40,000-$59,999, and 59% of participants were female. More detailed information about participant characteristics is reported in S8 Table in S1 File.

Beyond the typical socio-demographic variables, participants were also asked questions about stated consumption, attitudes towards climate change in general, and attitudes towards the links between climate change and beef and fuel consumption. A description of response options and frequency distributions for these questions are reported in S9 Table in S1 File.

Pearson correlation coefficients, and associated p-values testing the null hypothesis of no linear relationship between the covariates, are presented in S10-S12 Tables in S1 File. Coefficients greater than 0.3 are bolded. Education and income were moderately correlated with age (i.e., 0.308 and 0.427); however, there were no estimated coefficients greater than 0.3 between participant demographics and: stated consumption, or attitudes about climate change, or the links between climate change and beef and fuel consumption. There were also moderate relationships between personal importance of climate change and: humans causing climate change (0.438), willingness to eat less meat (0.317), and willingness to drive less (0.314). Moderate relationships were also estimated for responses to the willingness and effectiveness questions: effectiveness of eating less meat and driving less (0.317), effectiveness of eating less meat and willing to eat less meat (0.452), and willingness to eat less meat and drive less (0.428). It should be noted here that the estimated coefficient between effectiveness of driving less and willing to drive less was 0.249, compared to 0.452 for beef.

### Statistical tests

The null hypothesis is that the costs of mitigation per tCO_2_e via reducing beef consumption is equal to the costs of mitigation per tCO_2_e via limiting miles traveled. Participants provided offers for both behaviors, thus we can estimate within-subject differences in costs of mitigation. Matched data provide relatively more power than unmatched data because of reduction in the variation of unobservable characteristics across individuals. A sensitivity power analysis for a two-tail Wilcoxon signed-rank mean test using matched data with type I probability and power equal to 0.05 and 0.80, respectively, and our sample size (i.e., 626) can detect a very small effect size (i.e., 0.115).

To test the null hypothesis, we used two non-parametric tests: the Kolmogorov-Smirnov and Wilcoxon signed-rank tests. The Kolmogorov-Smirnov test was used to test the null hypothesis that the two samples are from the same distribution. While this test is informative about the overall distribution of estimated costs of mitigation, it does not incorporate the paired nature of how costs of mitigation for both behaviors were elicited. Thus, the Wilcoxon signed-rank test, a non-parametric approach, that accounts for repeated measures was also used to test the null hypothesis that the sample distributions are equal. Since costs of mitigation values are collected for five separate time horizons, it is necessary to conduct five Kolmogorov-Smirnov and Wilcoxon signed-rank tests (i.e., one test for each time horizon for each of the five time-horizons). A Bonferroni correction was used to account for the multiple comparisons between the behaviors across the five time-horizons.

The Kolmogorov-Smirnov and Wilcoxon signed-rank tests were also used to test the null hypothesis that costs of mitigation per tCO_2_e are the same across time horizons for each behavior (i.e., ten tests for both reducing beef consumption and limiting miles traveled). A Bonferroni correction was used again to account for the multiple comparisons between the time horizons within a behavior.

The tests described were estimated for the entire sample, and then again for plasticity targets of 25%, 50%, and 75%. Examining results for the varying plasticity targets provides the magnitude of abatement costs and potential for varying proportions of the sample engaged in a behavior change. The results focus on plasticity of 50% for two reasons: (1) it corresponds to the median cost in our sample, a value that allows us to minimize the influence of outlier offers and to make comparisons with published estimates of average abatement costs of other behaviors and policies; and (2) it corresponds to a reasonable policy target for achieving substantial reductions in GHG emissions. Results for plasticity targets of 25% and 75% are also presented, but in S1 File.

Finally, quantile regressions were estimated to examine heterogeneity in the amounts of compensation required to change a behavior across participant characteristics and additional covariates. Ordinary least squares regression models the conditional mean of the outcome variable, while quantile regression models specified conditional percentiles and is non-parametric so there are no assumptions implied by an error term [38]. Quantile regressions are commonly used in economic research [39] and allow us to model heterogeneity associated with the plasticity targets of 25%, 50%, and 75%.

## Results

Approximately 60% of participants reported eating beef weekly and 35% of participants reported driving 10,000 to 14,999 miles per year. Most participants, 64%, responded that climate change is mostly caused by human activities and 68% responded that climate change was either ‘very important’ or ‘extremely important.’ Interestingly, when asked to assess the effectiveness of the behaviors evaluated in this study, 60% of participants reported that driving less was ‘highly effective’ and 24% reported that eating less meat was ‘highly effective.’ Yet more participants reported being ‘certainly willing’ to eat less meat to combat climate change, 34% versus 22% for driving less.

The behavioral change with the most abatement potential—limiting personal vehicle use—is also the most expensive in our study population. Reducing GHG emissions via changes in beef consumption is likely more cost-effective than via changes in personal vehicle use. For the entire sample and for all time horizons, the within-subject private costs of mitigation per metric ton CO_2_-equivalent (tCO_2_e) for eliminating beef consumption are lower than the costs for carbon-equivalent limits on vehicle use (Wilcoxon signed-rank test; p-value <0.001 for every time horizon).

Also, we can reject the null hypothesis that the two empirical distributions for the private costs of mitigation per tCO_2_e are similar for all time horizons (Kolmogorov-Smirnov test; p-value <0.001 for every time horizon).

Table 1 reports the estimated abatement costs per person associated with a plasticity of approximately 50%. For eliminating beef consumption, the cost of mitigation per tCO_2_e over the various time horizons ranges from $642 to $798. For limiting vehicle use, the cost of mitigation per tCO_2_e ranges from $1,336 to $4,181. Thus, depending on the time horizon, GHG reductions from limiting vehicle use cost anywhere from roughly 1.8 to 6.5 times what equivalent reductions would cost from limiting beef consumption. In other words, for an equivalent amount of reduction in GHGs, adults in our sample required greater compensation for driving restrictions than for eliminating beef from their diets. Thus, policies targeted at reducing GHG emissions are likely more cost-effective for beef consumption compared to vehicle use.

**Table 1:** The estimated cost of voluntary change in beef consumption and personal vehicle use at a plasticity target of approximately 50%.

*Duration of Behavior Change*	Beef Consumption			Personal Vehicle Use			Cost of Mitigation		Cost Ratio
	Abatement Cost/Person ($US)	Plasticity (%)	Abatement Potential (tCO2e/person)	Abatement Cost/Person ($US)	Plasticity (%)	Abatement Potential (tCO2e/person)	Beef ($/tCO2e)	Vehicle ($/tCO2e)	Vehicle Beef
One Week	5	51	0.008	30	50	0.007	642	4,181	6.51
One Month	25	51	0.033	100	57	0.035	798	2,845	3.56
Three Months	75	52	0.102	200	53	0.098	734	2,040	2.79
Six Months	150	53	0.208	300	50	0.187	721	1,603	2.22
One Year	300	50	0.397	500	50	0.374	755	1,336	1.77

Note: Plasticity refers to the proportion of the public that would engage in the behavior at the corresponding abatement cost. Abatement Potential is the reduction in GHG emissions per person associated with the reported plasticity target.

Whereas the private costs of mitigation from eliminating beef consumption are similar across time horizons, the mitigation costs from limiting vehicle use decrease with longer time horizons. The mechanisms that cause this decrease across time horizons are unclear, as are the reasons why such mechanisms would be active for limiting vehicle use but not for beef consumption. One hypothesis is that well-established habits, like the use of cars, have behavioral momentum and are more difficult for individuals to change [40]. The high short-term private cost placed on limiting vehicle use can thus be viewed as a signal of a behavioral momentum of the status quo while the decrease in private mitigation costs over time signal the momentum of the new behavior in which driving has been limited. Thus, the temporal pattern of mitigation costs in an auction like ours may shed light on the temporal persistence of the targeted behavior change.

Policy-relevant plasticity targets for these behavior changes may be less than 50% [3, 37]. Thus, we also estimate abatement costs at a plasticity target of 25% (S1 Table in S1 File). At this value, the abatement cost ratios of the two behavior changes are smaller than they are at the plasticity target of 50%, particularly with longer horizons; the ratio is essentially equal to one for the one-year time horizon. But the magnitude of the abatement costs remains high for both behaviors: more than $235 per tCO_2_e.

We also estimate the costs at an ambitious plasticity target of 75% (S2 Table in S1 File). At this value, the abatement costs of limiting personal vehicle use are four or more times the costs of eliminating beef from one’s diet, and the magnitude of the abatement costs is greater than $1,720 per tCO_2_e.

These estimated costs are based on assumptions about the levels of beef consumption and driving in our sample in the absence of consumption limits. To explore how changing those assumptions affects our inferences, we change the status quo activity levels by plus or minus 5% and re-estimate the costs, which are represented by the shaded error bands in Figs 2 and 3 (S1–S5 Figs in S1 File show the abatement cost curves for the behaviors within each time horizon). Like the comparison of abatement costs in Table 1, a visual comparison of the curves implies that the costs are higher for limits in vehicle use (i.e., the vehicle curves are everywhere to the left of the beef curves). However, one can also see in these figures that the estimated costs of travel limits are more sensitive to changes in our assumptions about the status quo levels of driving than to changes in assumptions about the status quo level of beef consumption (because the limits placed on driving are relatively smaller than the limits placed on beef consumption). Yet even if the status quo driving level were 5% higher than we assumed, the estimated costs per tCO2e are still in the hundreds of dollars.

To illustrate heterogeneity in the amounts of compensation required to eliminate beef or limit vehicle use, we report quantile (i.e., median) regression coefficient estimates for all time-horizons in Tables 2 and 3. Here, quantile regressions are used to examine heterogeneity in compensation across participant socio-demographic characteristics, stated historical consumption, attitudes towards climate change, and attitudes towards the links between climate change and beef and fuel consumption.

**Table 2:** Estimated coefficients from a median regression examining the effects of participant characteristics and cost of voluntary change in beef consumption at a plasticity target of 50%.

*Variables*	One Week	One Month	Three Months	Six Months	One Year
Intercept	6.918	24.191	215.239***	409.587**	835.695**
	(5.546)	(22.808)	(75.323)	(165.354)	(369.196)
Annual Income	-0.444	-1.099	-1.722	6.273	6.194
	(0.297)	(1.096)	(4.160)	(9.627)	(21.215)
Annual Income—Prefer Not to Say	0.409	-2.085	-0.818	-5.405	31.927
	(2.533)	(8.379)	(26.694)	(48.515)	(111.936)
Age	0.908	1.093	2.081	9.827	31.194
	(0.560)	(1.508)	(5.491)	(13.180)	(26.972)
Education	-0.231	1.488	2.525	7.137	20.626
	(0.392)	(1.531)	(5.152)	(11.052)	(24.185)
Female	-2.124*	-11.044**	-22.291	-61.614**	-130.638*
	(1.116)	(4.392)	(14.278)	(30.671)	(67.977)
Household Size	-0.117	0.471	-2.718	-3.6121	0.821
	(0.384)	(1.487)	(4.535)	(11.182)	(23.376)
Black/African American	0.5161	8.599	8.990	-15.215	-30.999
	(2.536)	(8.920)	(33.170)	(75.604)	(188.123)
Hispanic, Latino, or Spanish Origin	-0.213	-12.686	-53.188	-179.472**	-270.262*
	(2.799)	(12.132)	(42.857)	(83.437)	(162.873)
White/Caucasian	-0.149	3.431	-7.420	-30.596	-9.151
	(1.268)	(5.467)	(16.092)	(38.585)	(82.008)
Suburban	1.505	5.904	-9.260	-4.461	-15.885
	(1.764)	(7.938)	(27.414)	(61.096)	(135.381)
Urban	3.013	1.149	-20.492	-66.733	-155.597
	(2.278)	(11.802)	(36.949)	(81.661)	(160.072)
Beef Consumption	3.072***	15.232***	40.036***	97.2973***	177.298***
	(0.515)	(2.640)	(8.506)	(19.730)	(44.580)
Cause of CC	1.560	2.683	1.3811	7.381	9.434
	(1.064)	(4.661)	(15.462)	(31.554)	(71.136)
Personal Importance of CC	0.915	2.859	1.089	-2.9187	3.508
	(0.568)	(2.520)	(7.121)	(17.225)	(42.550)
Effectiveness of Eating Less Meat to Combat CC	-0.449	-1.191	4.736	19.083	42.851
	(0.667)	(2.590)	(8.390)	(19.691)	(44.500)
Willingness to Eat Less Meat to Combat CC	-4.262***	-19.478***	-75.892***	-172.878***	-367.129***
	(0.725)	(2.630)	(8.958)	(19.013)	(43.888)

**Table 3:** Estimated coefficients from a median regression examining the effects of participant characteristics and cost of voluntary change in personal vehicle use at a plasticity target of 50%.

*Variables*	One Week	One Month	Three Months	Six Months	One Year
Intercept	67.787*	255.149**	702.769***	1131.883**	2392.458**
	(36.558)	(106.520)	(239.324)	(495.259)	(1069.585)
Annual Income	3.795*	8.055	25.873*	63.842**	146.289**
	(2.212)	(5.120)	(13.928)	(30.671)	(68.981)
Annual Income—Prefer Not to Say	0.644	23.781	77.722	92.054	162.948
	(8.617)	(36.647)	(61.447)	(136.102)	(245.814)
Age	0.929	2.400	-6.609	-34.687	-72.696
	(2.389)	(6.205)	(16.661)	(30.726)	(69.615)
Education	0.031	1.452	21.804	16.235	52.409
	(2.242)	(7.102)	(15.748)	(33.903)	(72.757)
Female	-13.933**	-49.020***	-151.067***	-348.770***	-683.194***
	(7.021)	(17.934)	(43.192)	(107.532)	(206.865)
Household Size	-0.021	2.350	16.841	40.693	93.8319
	(2.479)	(6.697)	(17.915)	(37.736)	(78.4542)
Black/African American	23.627	73.382	217.419	527.853**	1055.854*
	(27.471)	(65.817)	(134.628)	(254.098)	(626.756)
Hispanic, Latino, or Spanish Origin	-6.828	-20.987	-113.270	38.554	-474.757
	(19.660)	(42.347)	(140.505)	(293.167)	(517.288)
White/Caucasian	-5.145	-8.767	5.956	69.048	44.606
	(7.460)	(19.043)	(44.930)	(101.602)	(210.658)
Suburban	-11.425	-53.723	-183.577*	-357.879**	-303.202
	(11.495)	(32.809)	(96.034)	(156.897)	(343.322)
Urban	-14.044	-57.628	-241.952**	-471.211**	-644.773
	(14.046)	(43.925)	(122.876)	(229.726)	(461.816)
Fuel Consumption	23.387***	57.807***	168.152***	340.092***	662.979***
	(3.313)	(10.976)	(27.135)	(59.250)	(132.182)
Cause of CC	-3.099	-16.297	-86.048*	-120.443	-313.271
	(6.881)	(18.867)	(52.021)	(101.693)	(209.648)
Personal Importance of CC	0.620	6.515	31.110	50.597	115.748
	(3.375)	(9.783)	(22.455)	(45.607)	(117.007)
Effectiveness of Driving Less to Combat CC	-4.675	-29.108**	-104.124***	-160.204**	-462.921***
	(5.105)	(13.616)	(36.135)	(71.265)	(163.428)
Willingness to Driving Less to Combat CC	-18.720***	-50.739***	-141.465***	-236.039***	-482.277***
	(4.086)	(10.147)	(27.435)	(53.134)	(122.403)

As shown in S12 Table in S1 File, none of the socio-demographic characteristics are moderately associated with stated historical consumption or willingness to decrease consumption to combat climate change. Some socio-demographic characteristics are statistically associated with heterogeneity in compensation required for some time horizons and plasticity targets; however, statistical significance of coefficient estimates is inconsistent across time-horizons and plasticity targets. Female is the only socio-demographic characteristic that is statistically significant in most of the columns of Tables 2 and 3 and S3 through S6 Tables in S1 File. The coefficients have a negative sign, indicating that females, on average, required less compensation to change behavior, which may be a result of a relatively lower reliance on the targeted behaviors. In the US, self-reported estimates show that females consumed 40% less red meat than men from 2003 to 2004 [41] and drove 26% fewer miles from 2016 to 2017 [42]. Other socio-demographic characteristics are significant in a few columns of the tables. These results highlight the nuance associated with inducing behavioral changes across plasticity and time, not only across specific choices.

For both behaviors, there are only two statistically significant covariates across all plasticity targets and time horizons: stated historical consumption and willingness to decrease consumption to combat climate change. Required compensation to eliminate beef and limited vehicle use increases with stated consumption and decreases with stated wiliness to change behavior. Coefficient estimates for stated effectiveness of driving less to combat climate change and responding that climate change is mostly caused by humans were also significant in a few columns for the vehicle use tables. Thus, socio-demographic characteristics may not be useful for identifying segments of the public who are amenable to changing behavior in response to climate change.

## Discussion

Our results imply that it would cost at least $642 per tCO_2_e to reduce GHG emissions by inducing 50% of our study sample to eliminate beef consumption, and $1,336 per tCO_2_e to reduce emissions by inducing them to limit their personal vehicle use. The estimated magnitudes for the longer time horizons would not change even if we were to assume that the perceived costs of complying with the audit are at their maximum possible values. For example, even if we were to assume that the offers for the one-week horizon reflect only the perceived costs of experiencing the random audit, the estimated abatement costs for the one-year horizon would change by less than 5% (i.e., the change after subtracting the one-week horizon offers from the one-year horizon offers).

Even for a less ambitious goal of inducing 25% of the sample to eliminate beef or limit vehicle use, the costs for both behaviors would be more than $235 per tCO_2_e. These costs could be reduced by further lowering the target plasticity level, but lower levels would also yield far less GHG mitigation. An underlying lesson learned from the McKinsey Marginal Abatement Cost Curve [6] is that abatement costs are related to abatement potential (a.k.a., technical potential). That is, one can find low-cost mitigation options, including some with negative costs. Yet these options also typically have low abatement potential.

Our estimated abatement costs are higher than most estimates of the social cost of carbon (SCC), even at low plasticity targets. The SCC captures the marginal damage cost of emitting a tCO_2_e and varies based on assumptions (e.g., the social discount rate) and politics [43]. In the US, a median estimate of the SCC is $48 per tCO_2_e in 2018 dollars [44]. At that value, eliminating beef consumption or limiting vehicle use for a year would only make economic sense for about 20% of our sample. Our results are consistent with a recent meta-analysis [37], which reports low levels of behavioral plasticity for most household-level mitigation actions, and with a recent survey [45], which reports that low-carbon innovations in mobility and food are relatively unappealing to consumers.

Moreover, our estimated costs via changes in diet and travel are high in comparison to the estimated costs of reducing GHG through other means. For example, carbon offsets are sold to individuals and firms to fund projects that reduce GHG emissions (e.g., reforestation projects). Their prices are thus market-based measures for the cost of abating GHG emissions. Currently, the price to offset a tCO_2_e is between $10 to $13 [46–48]. Our median estimated abatement costs are also higher than all the lower-bound cost estimates reported in a study [49] that reviewed 50 economic studies of the abatement costs of policies (e.g., gasoline tax) and behavior changes (e.g., home energy use). Only the upper bound cost estimates for two policies were higher: solar photovoltaic subsidies ($2,100/ tCO_2_e,) and a low-carbon fuel standard ($2,900/ tCO_2_e). Our median abatement costs are also higher than the costs of removing atmospheric CO_2_ through enhanced rock weathering in the US ($160–190/tCO_2_e), although these estimates may not accurately reflect the risks associated with geo-engineering [50]. The differences in our estimated costs of reducing GHG via limiting travel and beef consumption and the estimated costs of reducing GHG via other means may arise because of differences in costs or estimation procedures. Clarity about the source of the differences can be had if future studies replicate an auction design with other GHG-reducing behaviors.

Nevertheless, we acknowledge that our estimated costs collected from a sample the Mid-Atlantic US may not generalize to other populations or to variations in our targeted behavior changes. For example, our estimated costs may have been different if we had asked people to limit beef consumption rather than eliminate it, or if we had extended the time horizon for limiting vehicle use. Moreover, the perceived costs of these behavior changes may decline with individual experience via habituation or the discovery of substitutes (i.e., learning by doing). As the behaviors become more prevalent in a society, the perceived costs may also decline via changes in social norms or via innovations that reduce the cost and availability of substitutes. Lastly, there is an ongoing debate about the atmospheric lifetime of CH4 compared to CO2, which could imply that our estimated costs associated with eliminating beef may be revised downwards in the future [51].

If, however, our study results approximately reflect how US adults perceive the costs associated with limiting beef consumption and driving, these behavior changes will not be easily achieved for a large proportion of the US population without substantial financial incentives, coercion, or major shifts in individual preferences. Alternatively, widespread behavior changes may be achievable through dramatic reductions in the private costs of behavior change. Thus, rather than try to limit current behaviors, governments may find that encouraging innovations that reduce the costs of behavior change, such as cheaper and more attractive meat alternatives and emissions-free vehicles, is a more cost-effective path forward both financially and politically. Also, institutions could use food service outlets to nudge food choice; for example, intentional designs of food options in a buffet-setting can influence the selection of meat [52], as can providing dietary information about food choice at the point of purchase [53]. Although, behavioral interventions targeted at reducing emissions typically have very small effects that do not persist after an intervention [54].

While our study uses revealed preference methods to measure the strength of preferences towards the behaviors of eating beef and driving a person vehicle, our approach does not account for substitutions that people likely make after reducing beef consumption and miles driving personal vehicle. To accurately measure the net effects on total food and travel emissions in field study would require extensive monitoring of individual behavior. Alternatively, a researcher could ask participants to forecast substitution behavior; however, this is likely an unreliable measure as substitution effects among possible substitutes (e.g., pork vs chicken) depend on changes in relative prices which would be impossible for participants to predict. Nevertheless, estimates from secondary data in the EU confirm that substitution effects will impact total food emissions [21, 22].

Future research examining wider dietary habits, and not only beef, could provide insight into possible GHG-reducing behavior across product categories and substation effects. Furthermore, it is not clear what caused the difference in estimated abatement costs between eliminating beef consumption and limiting vehicle use to decreases over longer-term horizons. Future work could focus on identifying mechanisms that affect the persistence of a behavioral momentum for high-emission choices.

## Supporting information

S1 File (DOCX)
