# Supplementary material for: Private costs of carbon emissions abatement by limiting beef consumption and vehicle use in the United States
Source: PLoS One. 2022 Jan 19;17(1):e0261372. doi: 10.1371/journal.pone.0261372 (PMC8769331; doi:10.1371/journal.pone.0261372)
Supplement: S1 File — (DOCX) [file pone.0261372.s001.docx]

**SUPPLEMENTARY INFORMATION**


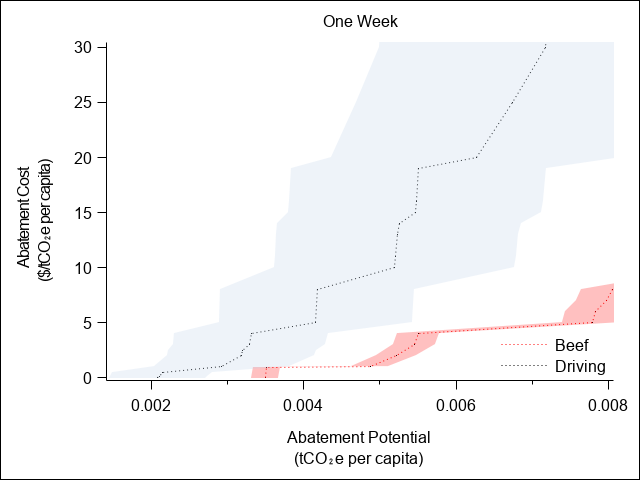


**Supplementary Figure 1. Revealed abatement cost curve to eliminate beef consumption and limit personal vehicle use for one week.** The lines represent the amount of money ($US) required to abate carbon emissions by eliminating beef consumption and limiting personal vehicle use. The figure presents cost curves for a plasticity target of approximately 50% (i.e., costs are a function of the offer values at or below the median offer). The error bands bound the potential changes in costs as the assumption about status quo driving levels changes by plus or minus 5%.


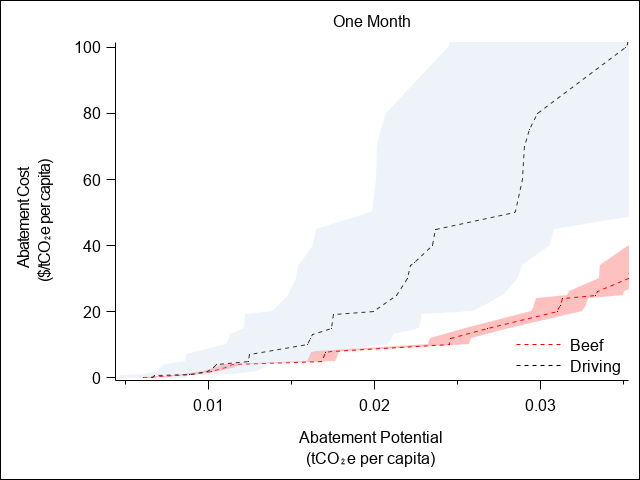


**Supplementary Figure 2. Revealed abatement cost curve to eliminate beef consumption and limit personal vehicle use for one month.** The lines represent the amount of money ($US) required to abate carbon emissions by eliminating beef consumption and limiting personal vehicle use. The figure presents cost curves for a plasticity target of approximately 50% (i.e., costs are a function of the offer values at or below the median offer). The error bands bound the potential changes in costs as the assumption about status quo driving levels changes by plus or minus 5%.


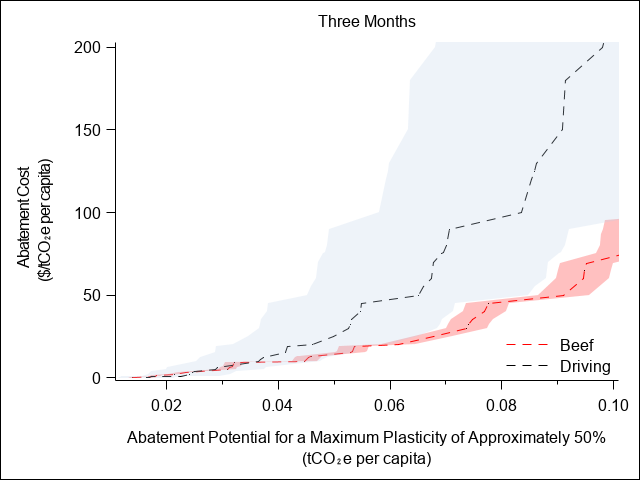


**Supplementary Figure 3. Revealed abatement cost curve to eliminate beef consumption and limit personal vehicle use for three months.** The lines represent the amount of money ($US) required to abate carbon emissions by eliminating beef consumption and limiting personal vehicle use. The figure presents cost curves for a plasticity target of approximately 50% (i.e., costs are a function of the offer values at or below the median offer). The error bands bound the potential changes in costs as the assumption about status quo driving levels changes by plus or minus 5%.


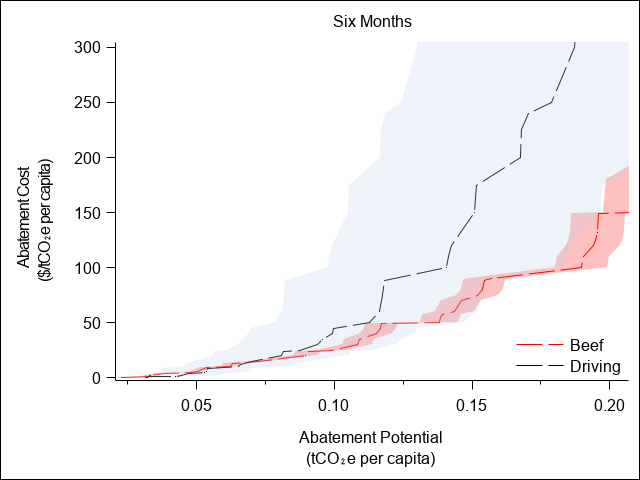


**Supplementary Figure 4. Revealed abatement cost curve to eliminate beef consumption and limit personal vehicle use for six months.** The lines represent the amount of money ($US) required to abate carbon emissions by eliminating beef consumption and limiting personal vehicle use. The figure presents cost curves for a plasticity target of approximately 50% (i.e., costs are a function of the offer values at or below the median offer). The error bands bound the potential changes in costs as the assumption about status quo driving levels changes by plus or minus 5%.


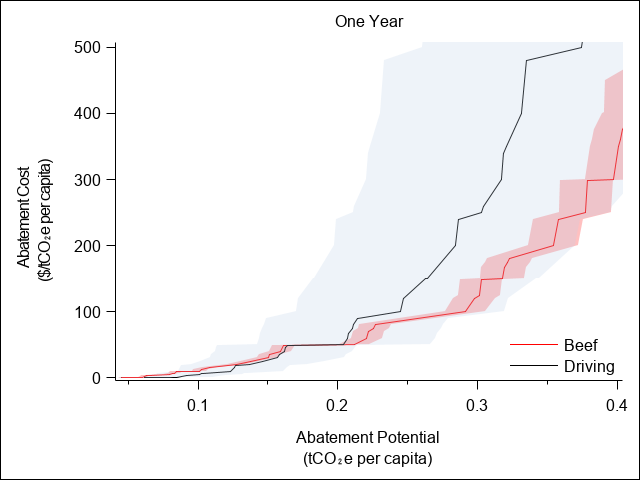


**Supplementary Figure 5. Revealed abatement cost curve to eliminate beef consumption and limit personal vehicle use for one year.** The lines represent the amount of money ($US) required to abate carbon emissions by eliminating beef consumption and limiting personal vehicle use. The figure presents cost curves for a plasticity target of approximately 50% (i.e., costs are a function of the offer values at or below the median offer). The error bands bound the potential changes in costs as the assumption about status quo driving levels changes by plus or minus 5%.

**Supplementary Table 1. The estimated cost of voluntary change in beef consumption and personal vehicle use at a plasticity target of approximately 25%.**

|  | Beef Consumption | | |  | Personal Vehicle Use | | |  | Cost of Mitigation | | Cost Ratio |
| --- | --- | --- | --- | --- | --- | --- | --- | --- | --- | --- | --- |
| *Duration of Behavior Change* | Abatement Cost/Person ($US) | Plasticity (%) | Abatement Potential  (tCO2e /person) |  | Abatement Cost/Person ($US) | Plasticity (%) | Abatement Potential  (tCO2e /person) |  | Beef  ($/tCO2e) | Vehicle  ($/tCO2e) | Vehicle Beef |
| One Week | 0.94 | 23 | 0.004 |  | 4 | 23 | 0.003 |  | 267 | 1,207 | 4.52 |
| One Month | 5 | 26 | 0.017 |  | 10 | 26 | 0.016 |  | 297 | 627 | 2.11 |
| Three Months | 13 | 23 | 0.046 |  | 20 | 25 | 0.046 |  | 282 | 434 | 1.54 |
| Six Months | 25 | 25 | 0.099 |  | 30 | 25 | 0.095 |  | 252 | 320 | 1.27 |
| One Year | 50 | 27 | 0.212 |  | 50 | 27 | 0.204 |  | 236 | 245 | 1.04 |

**Supplementary Table 2. The estimated cost of voluntary change in beef consumption and personal vehicle use at a plasticity target** **of approximately 75%.**

|  | Beef Consumption | | |  | Personal Vehicle Use | | |  | Cost of Mitigation | | Cost Ratio |
| --- | --- | --- | --- | --- | --- | --- | --- | --- | --- | --- | --- |
| *Duration of Behavior Change* | Abatement Cost/Person ($US) | Plasticity (%) | Abatement Potential  (tCO2e /person) |  | Abatement Cost/Person ($US) | Plasticity (%) | Abatement Potential  (tCO2e /person) |  | Beef  ($/tCO2e) | Vehicle  ($/tCO2e) | Vehicle Beef |
| One Week | 20 | 75 | 0.011 |  | 100 | 76 | 0.011 |  | 1,747 | 9,175 | 5.25 |
| One Month | 80 | 71 | 0.046 |  | 450 | 75 | 0.046 |  | 1,722 | 9,711 | 5.64 |
| Three Months | 250 | 73 | 0.143 |  | 1,000 | 75 | 0.140 |  | 1,747 | 7,147 | 4.09 |
| Six Months | 600 | 76 | 0.301 |  | 2,400 | 75 | 0.280 |  | 1,990 | 8,577 | 4.31 |
| One Year | 1,200 | 75 | 0.595 |  | 5,000 | 76 | 0.567 |  | 2,016 | 8,822 | 4.38 |

**Supplementary Table 3. Estimated coefficients from a percentile regression examining the effects of participant characteristics and cost of voluntary change in beef consumption at a plasticity** target **of 25%.**

|  | One Week | One Month | Three Months | Six Months | One Year |
| --- | --- | --- | --- | --- | --- |
| Intercept | 4.763* | 10.905 | 48.159 | 52.348 | 170.241 |
|  | (2.799) | (10.347) | (37.546) | (54.504) | (115.284) |
| Annual Income | 0.070 | -0.284 | 0.699 | 0.753 | -3.742 |
|  | (0.143) | (0.489) | (1.896) | (2.944) | (7.224) |
| Annual Income –  Prefer Not to Say | 0.0643 | 1.235 | -0.325 | 3.250 | 15.971 |
|  | (0.537) | (2.415) | (6.218) | (9.542) | (27.116) |
| Age | 0.053 | 0.676 | 1.54 | 2.011 | 10.989 |
|  | (0.173) | (0.817) | (2.220) | (4.174) | (8.046) |
| Education | 0.015 | 0.131 | 1.190 | 0.562 | 2.115 |
|  | (0.190) | (0.649) | (2.399) | (4.017) | (9.302) |
| Female | -1.183** | -3.641* | -9.762* | -28.319*** | -71.701*** |
|  | (0.508) | (1.909) | (5.876) | (10.112) | (19.646) |
| Household Size | -0.045 | -0.186 | -1.763 | -4.350 | -6.726 |
|  | (0.202) | (0.711) | (2.402) | (3.988) | (9.069) |
| Black/African American | -0.3305 | 1.476 | 5.779 | 15.099 | 32.175 |
|  | (1.020) | (4.128) | (14.791) | (22.850) | (54.002) |
| Hispanic, Latino, or  Spanish Origin | 0.313 | -1.072 | -10.250 | -15.882 | -27.572 |
|  | (1.427) | (3.954) | (12.607) | (22.954) | (53.420) |
| White/Caucasian | 0.065 | 0.873 | 7.467 | 18.4475 | 34.513 |
|  | (0.600) | (2.226) | (7.472) | (11.535) | (29.245) |
| Suburban | -1.868* | -3.524 | -14.275 | -0.423 | -76.404 |
|  | (1.051) | (4.031) | (12.611) | (21.593) | (62.332) |
| Urban | -2.142 | -7.678 | -27.535* | -35.300 | -161.076** |
|  | (1.329) | (4.720) | (14.771) | (25.862) | (72.479) |
| Beef Consumption | 0.862** | 5.436*** | 14.663*** | 31.310*** | 68.295*** |
|  | (0.336) | (1.280) | (4.598) | (6.700) | (14.490) |
| Cause of CC | -0.237 | -1.633 | -4.334 | -5.570 | -15.258 |
|  | (0.550) | (2.099) | (6.820) | (10.717) | (21.668) |
| Personal Importance of CC | 0.294 | 1.482 | 0.925 | 7.117 | 16.778 |
|  | (0.295) | (1.093) | (3.550) | (6.499) | (14.181) |
| Effectiveness of Eating Less Meat to Combat CC | -0.404 | -0.883 | -0.164 | -1.002 | 0.192 |
|  | (0.332) | (1.200) | (3.622) | (6.576) | (16.278) |
| Willingness to Eat Less Meat to Combat CC | -1.067*** | -5.118*** | -17.954*** | -33.925*** | -74.257*** |
|  | (0.337) | (1.047) | (3.568) | (5.981) | (14.325) |

**Supplementary Table 4. Estimated coefficients from a percentile regression examining the effects of participant characteristics and cost of voluntary change in personal vehicle use at a plasticity** target **of 25%.**

|  | One Week | One Month | Three Months | Six Months | One Year |
| --- | --- | --- | --- | --- | --- |
| Intercept | 12.831 | 65.630* | 140.289* | 215.230 | 376.635 |
|  | (11.865) | (35.615) | (75.724) | (141.052) | (230.446) |
| Annual Income | 1.671** | 0.916 | 7.151 | 24.994** | 30.597* |
|  | (0.703) | (2.129) | (4.526) | (9.849) | (16.688) |
| Annual Income –  Prefer Not to Say | 3.263 | 14.240* | 16.691 | 22.350 | 64.657 |
|  | (2.579) | (7.775) | (22.180) | (38.163) | (73.187) |
| Age | 0.277 | 4.197* | 0.210 | -11.614 | -11.743 |
|  | (0.739) | (2.254) | (5.371) | (9.068) | (14.708) |
| Education | 0.964 | 1.247 | 0.478 | 1.495 | 0.483 |
|  | (0.792) | (2.080) | (5.059) | (8.398) | (13.653) |
| Female | -1.276 | -12.440** | -27.972** | -40.351** | -120.459** |
|  | (1.925) | (5.796) | (13.472) | (20.400) | (48.634) |
| Household Size | 0.636 | 2.077 | 2.448 | 4.834 | 13.439 |
|  | (0.912) | (2.348) | (6.077) | (10.115) | (18.466) |
| Black/African American | 13.544** | 44.166*** | 83.539** | 146.086*** | 256.391** |
|  | (6.476) | (10.510) | (38.357) | (54.329) | (111.897) |
| Hispanic, Latino, or  Spanish Origin | -1.893 | 8.070 | 18.701 | 15.259 | 36.848 |
|  | (7.815) | (17.859) | (33.236) | (61.722) | (101.791) |
| White/Caucasian | 0.670 | 2.531 | 10.880 | 16.288 | 28.291 |
|  | (2.215) | (7.100) | (16.488) | (24.041) | (52.580) |
| Suburban | -6.165 | -18.153 | -28.549 | -47.731 | -95.234 |
|  | (4.290) | (11.432) | (22.877) | (34.786) | (79.972) |
| Urban | -11.937* | -39.639*** | -80.396** | -110.508** | -237.766** |
|  | (6.133) | (14.641) | (35.049) | (55.574) | (112.261) |
| Fuel Consumption | 7.201*** | 25.672*** | 52.906*** | 90.044*** | 170.218*** |
|  | (1.075) | (3.730) | (9.420) | (15.534) | (31.555) |
| Cause of CC | -4.279** | -12.960** | -27.016* | -48.516** | -84.683* |
|  | (1.768) | (5.624) | (14.891) | (26.624) | (50.237) |
| Personal Importance of CC | 0.471 | 2.040 | 3.652 | 2.146 | 17.947 |
|  | (1.154) | (3.001) | (7.354) | (11.993) | (21.785) |
| Effectiveness of Driving Less to Combat CC | -2.508 | -10.362** | -21.129* | -38.5512* | -67.190* |
|  | (1.762) | (4.540) | (12.563) | (22.669) | (34.857) |
| Willingness to Driving Less to Combat CC | -5.072*** | -18.418*** | -34.857*** | -49.705*** | -92.756*** |
|  | (1.195) | (3.481) | (8.487) | (13.348) | (25.327) |

**Supplementary Table 5. Estimated coefficients from a percentile regression examining the effects of participant characteristics and cost of voluntary change in beef consumption at a plasticity** target **of 75%.**

|  | One Week | One Month | Three Months | Six Months | One Year |
| --- | --- | --- | --- | --- | --- |
| Intercept | 47.878*** | 179.731*** | 538.015*** | 842.352* | 4795.892*** |
|  | (15.261) | (66.145) | (206.177) | (452.429) | (1222.549) |
| Annual Income | -0.033 | -1.884 | -8.216 | -22.432 | 1.718 |
|  | (1.046) | (3.401) | (8.892) | (20.366) | (56.859) |
| Annual Income –  Prefer Not to Say | 2.446 | 49.024 | 68.138 | 118.0622 | 89.773 |
|  | (7.960) | (36.680) | (99.546) | (279.112) | (357.624) |
| Age | 2.640* | 4.825 | 24.713 | 31.273 | -10.515 |
|  | (1.449) | (5.428) | (18.361) | (36.909) | (84.848) |
| Education | -1.102 | 4.380 | 16.542 | 56.164* | 94.649 |
|  | (1.401) | (4.312) | (15.120) | (30.104) | (81.233) |
| Female | -8.685** | -46.434*** | -184.876*** | -404.022*** | -880.441*** |
|  | (3.710) | (14.312) | (46.583) | (106.865) | (287.754) |
| Household Size | 0.010 | 1.138 | -5.000 | -14.934 | -30.007 |
|  | (1.076) | (3.956) | (13.223) | (30.690) | (75.502) |
| Black/African American | -3.701 | 5.209 | -6.775 | -15.560 | -63.9061 |
|  | (12.410) | (31.641) | (91.060) | (167.223) | (431.125) |
| Hispanic, Latino, or  Spanish Origin | -6.001 | -10.549 | -104.484 | -277.522** | -554.977 |
|  | (8.333) | (25.198) | (70.881) | (135.832) | (440.226) |
| White/Caucasian | -7.199* | -5.949 | -57.549 | -99.068 | -174.643 |
|  | (4.364) | (13.468) | (51.378) | (90.574) | (280.150) |
| Suburban | 1.084 | -4.855 | 63.243 | 53.980 | -256.575 |
|  | (5.862) | (23.113) | (57.034) | (142.591) | (458.402) |
| Urban | -1.120 | -17.132 | 29.882 | -53.297 | -475.865 |
|  | (6.966) | (28.572) | (75.536) | (141.384) | (536.893) |
| Beef Consumption | 5.730*** | 24.476*** | 90.758*** | 207.989*** | 395.579*** |
|  | (1.377) | (5.213) | (19.050) | (37.844) | (102.047) |
| Cause of CC | 0.777 | 10.285 | 0.201 | 66.153 | -93.743 |
|  | (3.255) | (13.780) | (46.505) | (122.562) | (205.513) |
| Personal Importance of CC | 0.737 | -8.212 | -19.984 | -47.397 | -134.828 |
|  | (1.711) | (6.075) | (21.522) | (54.729) | (128.065) |
| Effectiveness of Eating Less Meat to Combat CC | 0.765 | 6.530 | 10.583 | 33.186 | 20.359 |
|  | (1.980) | (5.735) | (20.543) | (50.109) | (121.491) |
| Willingness to Eat Less Meat to Combat CC | -13.036*** | -50.165*** | -148.711*** | -253.64*** | -1009.910*** |
|  | (2.119) | (9.198) | (30.119) | (68.653) | (177.334) |

**Supplementary Table 6. Estimated coefficients from a percentile regression examining the effects of participant characteristics and cost of voluntary change in personal vehicle use at a plasticity** target **of 75%.**

|  | One Week | One Month | Three Months | Six Months | One Year |
| --- | --- | --- | --- | --- | --- |
| Intercept | 144.289 | 395.136 | 560.840 | 2522.474 | 4795.892*** |
|  | (103.182) | (375.263) | (1190.614) | (2271.291) | (1222.549) |
| Annual Income | 9.406 | 6.302 | 7.861 | 113.863 | 1.718 |
|  | (6.303) | (24.341) | (59.165) | (118.765) | (56.859) |
| Annual Income –  Prefer Not to Say | 14.601 | 11.207 | 71.923 | -91.382 | 89.773 |
|  | (74.084) | (144.225) | (360.050) | (906.809) | (357.624) |
| Age | -4.393 | -23.943 | -62.213 | -145.497 | -10.515 |
|  | (9.264) | (27.346) | (68.556) | (152.886) | (84.848) |
| Education | -2.129 | 45.286* | 81.968 | 196.147 | 94.649 |
|  | (7.427) | (26.671) | (67.356) | (147.706) | (81.233) |
| Female | -15.562 | -156.119* | -416.943* | -991.747** | -880.44*** |
|  | (20.432) | (81.147) | (236.607) | (395.235) | (287.754) |
| Household Size | 1.080 | 34.842 | 88.298 | 133.824 | -30.007 |
|  | (9.506) | (26.966) | (89.253) | (169.182) | (75.502) |
| Black/African American | 108.786 | 183.310 | 289.266 | 648.648 | -63.906 |
|  | (166.151) | (247.331) | (709.778) | (1980.316) | (431.125) |
| Hispanic, Latino, or  Spanish Origin | 31.054 | 206.819 | 363.918 | 760.903 | -554.977 |
|  | (58.554) | (267.865) | (729.932) | (1247.081) | (440.226) |
| White/Caucasian | 10.872 | 24.0868 | 2.284 | -41.990 | -174.643 |
|  | (19.804) | (91.316) | (253.221) | (555.512) | (280.150) |
| Suburban | -27.860 | -108.585 | -304.410 | -301.402 | -256.575 |
|  | (47.034) | (125.078) | (342.760) | (577.949) | (458.402) |
| Urban | -17.045 | -202.915 | -481.053 | -981.331 | -475.865 |
|  | (56.206) | (145.267) | (419.879) | (731.207) | (536.893) |
| Fuel Consumption | 82.052*** | 223.116*** | 784.919*** | 1501.326*** | 395.579*** |
|  | (11.302) | (40.1694) | (115.734) | (202.035) | (102.047) |
| Cause of CC | 28.445 | 42.641 | 99.566 | -22.552 | -93.743 |
|  | (26.750) | (81.419) | (230.303) | (460.987) | (205.513) |
| Personal Importance of CC | -8.322 | 15.797 | 11.711 | -97.966 | -134.828 |
|  | (13.057) | (39.452) | (116.870) | (239.002) | (128.065) |
| Effectiveness of Driving Less to Combat CC | -7.077 | -64.453 | -68.732 | -485.495* | 20.359 |
|  | (17.634) | (61.399) | (156.534) | (280.575) | (121.491) |
| Willingness to Driving Less to Combat CC | -52.391*** | -151.905*** | -341.120*** | -537.973** | -1009.910*** |
|  | (12.486) | (44.746) | (125.199) | (267.314) | (177.334) |

**Supplementary Table 7. Values used to determine the mileage caps**

| Beef Emissions | |  | Personal Vehicle Emissions | |  | Difference in Emissions | |
| --- | --- | --- | --- | --- | --- | --- | --- |
| Average Disappearance^*^ | 37.06 kg/year  (USDA) |  | Average Miles Driven | 13,476 miles/year  (USDOTa) |  | Average Annual Fuel GHG Emissions | 4522.92 kgCO_2_e |
| Direct Emissions | 21.3 kgCO_2_e per kg  (Rotz et al. 2019;  Avery and Avery 2008) |  | Average Miles Traveled in State Where the Study Occurred | 11,657 miles/year  (USDOTb) |  | Average Annual Beef GHG Emissions | 789.38 kgCO_2_e |
|  |  |  | Average of Miles Driven and Traveled^*^ | 12,567 miles |  | Difference in Annual GHG Emissions | 3,733.54 kgCO_2_e |
| Average Annual GHG Emissions | 789.38 kgCO_2_e |  | Average Fuel Economy | 24.7 miles per gal  (Hula et al. 2018) |  | Miles allowed to travel | 10,373.29 miles/year |
|  |  |  | Average Fuel Use | 508.77 gal/year |  |  |  |
|  |  |  | Direct Emissions^34^ | 8.89 kgCO2e per gal  (EIA) |  |  |  |
|  |  |  | Average Annual  GHG Emissions | 4522.92 kgCO_2_e |  |  |  |

* “Disappearance” is the amount used in domestic markets, including fresh and processed meat sold through grocery stores and used in restaurants. It represents the best measure of average annual consumption per capita. “Disappearance” and “Average miles driven and traveled” are the two assumptions that are varied in the sensitivity analyses that generate the error bars in Figures 1-2 and Supplementary Figures 1-5.

**Supplementary Table 8. Further details about participant characteristics.**

| Characteristic | Proportion |
| --- | --- |
| Age |  |
| 18-24 years | 36.10 |
| 25-34 years | 21.88 |
| 35-44 years | 16.77 |
| 45-54 years | 10.22 |
| 55-64 years | 9.90 |
| 65-74 years | 3.99 |
| 75 years or older | 1.12 |
| Education |  |
| Some High School | 3.99 |
| High School Diploma / GED | 23.80 |
| Some College | 5.43 |
| Associates Degree | 38.50 |
| Bachelor's Degree | 19.97 |
| Master’s Degree | 8.31 |
| Ph.D. / Professional Degree | 3.99 |
| Sex |  |
| Female | 59.42 |
| Male | 39.94 |
| Prefer Not to Say | 0.64 |
| Household Size |  |
| One | 9.42 |
| Two | 24.28 |
| Three | 23.96 |
| Four | 25.72 |
| Five or more | 16.61 |
| Annual Income |  |
| Under $20,000 | 27.32 |
| $20,000-$39,999 | 15.18 |
| $40,000-$59,999 | 11.82 |
| $60,000-$79,999 | 11.98 |
| $80,000-$99,999 | 8.15 |
| $100,000-$119,999 | 5.27 |
| $120,000-$139,999 | 3.04 |
| $140,000-$159,999 | 2.56 |
| $160,000 or more | 4.79 |
| Prefer Not to Say | 9.90 |
| Race |  |
| Black/African American | 5.59 |
| Hispanic, Latino, or Spanish Origin | 3.67 |
| White/Caucasian | 71.73 |
| Multiracial/Other | 26.25 |
| Living Density |  |
| Suburban | 77.00 |
| Rural | 9.74 |
| Urban | 13.26 |

**Supplementary Table 9. Further details about additional covariates.**

| Covariate | Proportion |
| --- | --- |
| Beef Consumption |  |
| Never | 2.24 |
| Several times a year | 8.79 |
| Monthly | 24.60 |
| Weekly | 59.58 |
| Daily | 4.79 |
| Vehicle Use |  |
| Less than 5,000 miles | 17.25 |
| 5,000-9,999 miles | 27.80 |
| 10,000-14,999 miles | 34.98 |
| 15,000-19,999 miles | 12.46 |
| 20,000 miles or more | 7.51 |
| Cause of Climate Change |  |
| Caused mostly by human activities | 64.38 |
| Caused about equally by human activities and natural changes | 27.16 |
| Caused mostly by natural changes in the environment | 5.43 |
| None of the above because climate change isn't happening | 0.96 |
| I don't know | 2.08 |
| Personal Importance of Climate Change |  |
| Not at all important | 2.56 |
| Slightly important | 5.27 |
| Moderately important | 23.80 |
| Very important | 35.62 |
| Extremely important | 32.75 |
| Effectiveness of Eating Less Meat to Combat Climate Change |  |
| Not effective at all | 10.06 |
| Not very effective | 28.59 |
| Effective | 32.91 |
| Highly effective | 24.28 |
| I don't know | 4.15 |
| Effectiveness of Driving Less to Combat Climate Change |  |
| Not effective at all | 2.40 |
| Not very effective | 5.27 |
| Effective | 29.55 |
| Highly effective | 60.22 |
| I don't know | 2.56 |
| Willingness to Eat Less Meat to Combat Climate Change |  |
| Certainly not willing | 8.15 |
| Likely not willing | 20.77 |
| Likely willing | 35.78 |
| Certainly willing | 34.35 |
| I don't know | 0.96 |
| Willingness to Driving Less to Combat Climate Change |  |
| Certainly not willing | 8.31 |
| Likely not willing | 32.27 |
| Likely willing | 36.90 |
| Certainly willing | 21.57 |
| I don't know | 0.96 |

**Supplementary Table 10. Estimated correlation coefficients between participant characteristics.**

|  | Age | Female | White / Caucasian | Black / African American | Hispanic, Latino, or Spanish Origin | Suburban | Urban | Household Size | Education |
| --- | --- | --- | --- | --- | --- | --- | --- | --- | --- |
| Age | 1 |  |  |  |  |  |  |  |  |
|  |  |  |  |  |  |  |  |  |  |
| Female | -0.041 | 1 |  |  |  |  |  |  |  |
|  | 0.303 |  |  |  |  |  |  |  |  |
| White / Caucasian | 0.121 | -0.006 | 1 |  |  |  |  |  |  |
|  | 0.002 | 0.883 |  |  |  |  |  |  |  |
| Black / African American | -0.002 | -0.011 | **-0.388** | 1 |  |  |  |  |  |
|  | 0.970 | 0.778 | <.0001 |  |  |  |  |  |  |
| Hispanic, Latino, or Spanish Origin | 0.005 | 0.0230 | **-0.311** | -0.048 | 1 |  |  |  |  |
|  | 0.900 | 0.565 | <.0001 | 0.235 |  |  |  |  |  |
| Suburban | 0.008 | 0.059 | 0.045 | -0.032 | -0.014 | 1 |  |  |  |
|  | 0.835 | 0.144 | 0.266 | 0.421 | 0.721 |  |  |  |  |
| Urban | -0.019 | -0.080 | -0.100 | 0.069 | 0.024 | -0.715 | 1 |  |  |
|  | 0.627 | 0.046 | 0.013 | 0.085 | 0.552 | <.0001 |  |  |  |
| Household Size | -0.217 | -0.010 | -0.092 | 0.0930 | 0.051 | 0.055 | -0.100 | 1 |  |
|  | <.0001 | 0.801 | 0.021 | 0.020 | 0.205 | 0.171 | 0.012 |  |  |
| Education | **0.308** | 0.004 | -0.001 | -0.021 | -0.116 | -0.017 | 0.034 | -0.245 | 1 |
|  | <.0001 | 0.915 | 0.983 | 0.602 | 0.004 | 0.676 | 0.402 | <.0001 |  |
| Income | **0.427** | -0.074 | 0.064 | -0.086 | -0.012 | 0.015 | -0.034 | -0.062 | 0.296 |
|  | <.0001 | 0.064 | 0.108 | 0.031 | 0.756 | 0.701 | 0.392 | 0.122 | <.0001 |
| Note: P-Values are reported below the Pearson correlation coefficient, testing the null hypothesis that a linear relationship between two variables is equal to zero. | | | | | | | | | |

**Supplementary Table 11. Estimated correlation coefficients between additional covariates.**

|  | Beef Consumption | Vehicle Use | Cause of CC | Personal Importance of CC | Effectiveness of Eating Less Meat to Combat CC | Effectiveness of Driving Less to Combat CC | Willingness to Eat Less Meat to Combat CC |
| --- | --- | --- | --- | --- | --- | --- | --- |
| Beef Consumption | 1 |  |  |  |  |  |  |
|  |  |  |  |  |  |  |  |
| Vehicle Use | 0.091 | 1 |  |  |  |  |  |
|  | 0.023 |  |  |  |  |  |  |
| Cause of CC | 0.123 | 0.094 | 1 |  |  |  |  |
|  | 0.002 | 0.018 |  |  |  |  |  |
| Personal Importance of CC | -0.068 | -0.112 | **-0.438** | 1 |  |  |  |
|  | 0.087 | 0.005 | <.0001 |  |  |  |  |
| Effectiveness of Eating Less Meat to Combat CC | -0.137 | -0.048 | -0.283 | 0.400 | 1 |  |  |
|  | 0.001 | 0.229 | <.0001 | <.0001 |  |  |  |
| Effectiveness of Driving Less to Combat CC | -0.047 | -0.018 | -0.294 | 0.296 | **0.317** | 1 |  |
|  | 0.244 | 0.649 | <.0001 | <.0001 | <.0001 | <.0001 |  |
| Willingness to Eat Less Meat to Combat CC | -0.265 | -0.057 | -0.234 | **0.317** | **0.452** | 0.257 | 1 |
|  | <.0001 | 0.152 | <.0001 | <.0001 | <.0001 | <.0001 | <.0001 |
| Willingness to Driving Less to Combat CC | -0.108 | -0.246 | -0.219 | **0.314** | 0.204 | 0.249 | **0.428** |
|  | 0.007 | <.0001 | <.0001 | <.0001 | <.0001 | <.0001 | <.0001 |
| Note: P-Values are reported below the Pearson correlation coefficient, testing the null hypothesis that a linear relationship between two variables is equal to zero. | | | | | | | |

**Supplementary Table 12. Estimated correlation coefficients between participant characteristics and additional covariates.**

|  | Age | Female | White / Caucasian | Black / African American | Hispanic, Latino, or Spanish Origin | Suburban | Urban | Household Size | Education | Income | |
| --- | --- | --- | --- | --- | --- | --- | --- | --- | --- | --- | --- |
| Beef Consumption | 0.119 | -0.173 | 0.136 | -0.082 | 0.054 | -0.106 | 0.044 | -0.020 | 0.013 | 0.020 | |
|  | 0.003 | <.0001 | 0.001 | 0.039 | 0.177 | 0.008 | 0.269 | 0.620 | 0.755 | 0.614 | |
| Vehicle Use | 0.150 | -0.108 | 0.127 | -0.017 | -0.075 | -0.051 | 0.008 | 0.013 | 0.108 | 0.167 | |
|  | <.0001 | 0.007 | 0.002 | 0.665 | 0.060 | 0.203 | 0.843 | 0.742 | 0.007 | <.0001 | |
| Cause of CC | 0.17388 | -0.092 | -0.017 | 0.024 | 0.028 | -0.066 | 0.030 | -0.017 | 0.010 | 0.099 | |
|  | <.0001 | 0.022 | 0.671 | 0.554 | 0.485 | 0.100 | 0.457 | 0.671 | 0.811 | 0.013 | |
| Personal Importance of CC | -0.096 | 0.110 | -0.037 | -0.012 | 0.061 | 0.041 | 0.017 | -0.045 | -0.010 | -0.091 | |
|  | 0.016 | 0.006 | 0.356 | 0.760 | 0.130 | 0.312 | 0.664 | 0.259 | 0.801 | 0.023 | |
| Effectiveness of Eating Less Meat to Combat CC | -0.141 | 0.075 | -0.050 | -0.016 | 0.030 | 0.036 | 0.034 | 0.050 | -0.068 | -0.057 | |
|  | <.0001 | 0.059 | 0.213 | 0.693 | 0.447 | 0.371 | 0.401 | 0.211 | 0.087 | 0.157 | |
| Effectiveness of Driving Less to Combat CC | -0.175 | 0.103 | -0.010 | 0.016 | -0.031 | 0.070 | -0.057 | -0.024 | -0.068 | -0.058 | |
|  | <.0001 | 0.01 | 0.795 | 0.700 | 0.437 | 0.081 | 0.158 | 0.548 | 0.088 | 0.145 | |
| Willingness to Eat Less Meat to Combat CC | -0.010 | 0.184 | 0.017 | 0.060 | 0.019 | 0.107 | -0.012 | -0.050 | 0.006 | 0.020 | |
|  | 0.804 | <.0001 | 0.671 | 0.133 | 0.628 | 0.008 | 0.774 | 0.208 | 0.887 | 0.617 | |
| Willingness to Driving Less to Combat CC | 0.020 | 0.101 | -0.058 | -0.008 | 0.063 | 0.060 | 0.011 | -0.013 | 0.027 | 0.009 | |
|  | 0.613 | 0.012 | 0.149 | 0.834 | 0.114 | 0.137 | 0.790 | 0.755 | 0.503 | 0.831 | |
| Note: P-Values are reported below the Pearson correlation coefficient, testing the null hypothesis that a linear relationship between two variables is equal to zero. | | | | | | | | | | |  |
